# Non-Steroidal Anti-Inflammatory Drugs and Aspirin Therapy for the Treatment of Acute and Recurrent Idiopathic Pericarditis

**DOI:** 10.3390/ph9020017

**Published:** 2016-03-23

**Authors:** Nicholas Schwier, Nicole Tran

**Affiliations:** 1 Department of Pharmacy, Clinical and Administrative Sciences, College of Pharmacy, University of Oklahoma, 1110 N. Stonewall Avenue, CPB 214, Oklahoma City, OK 73117, USA; 2 Department of Cardiology, College of Medicine, University of Oklahoma, 825 N.E. 10th Street, 2E, Oklahoma City, OK 74104, USA; Nicole-Tran@ouhsc.edu

**Keywords:** idiopathic, pericarditis, non-steroidal anti-inflammatory drugs (NSAIDs), aspirin (ASA)

## Abstract

Aspirin (ASA) and non-steroidal anti-inflammatory drugs (NSAIDs) are a mainstay of therapy for the treatment of idiopathic pericarditis (IP). A comprehensive review consisting of pertinent clinical literature, pharmacokinetic, and pharmacodynamic considerations, has not been released in recent years. This review will facilitate the clinician’s understanding of pharmacotherapeutic considerations for using ASA/NSAIDs to treat IP. Data were compiled using clinical literature consisting of case reports, cohort data, retrospective and prospective studies, and manufacturer package inserts. ASA, ibuprofen, indometacin, and ketorolac relatively have the most evidence in the treatment of IP, provide symptomatic relief of IP, and should be tapered accordingly. ASA is the drug of choice in patients with coronary artery disease (CAD), heart failure (HF), or renal disease, but should be avoided in patients with asthma and nasal polyps, who are naïve to ASA therapy. Ibuprofen is an inexpensive and relatively accessible option in patients who do not have concomitant CAD, HF, or renal disease. Indometacin is not available over-the-counter in the USA, and has a relatively higher incidence of central nervous system (CNS) adverse effects. Ketorolac is an intravenous option; however, clinicians must be mindful of the maximum dose that can be administered. While ASA/NSAIDs do not ameliorate the disease process of IP, they are part of first-line therapy (along with colchicine), for preventing recurrence of IP. ASA/NSAID choice should be dictated by comorbid conditions, tolerability, and adverse effects. Additionally, the clinician should be mindful of considerations such as tapering, high-sensitivity CRP monitoring, bleeding risk, and contraindications to ASA/NSAID therapy.

## 1. Introduction

Pericarditis is the inflammation of the pericardium, the membranous sac which envelops the heart [1]. Without the pericardium, the heart would be ill-protected from overfilling, traumatic processes, and infection [1]. The incidence of patients hospitalized for acute pericarditis is estimated to be 3.32/100,000 person-years and it is the most common disease of the pericardium witnessed in practice [2,3]. Pericarditis is the cause of 5% of emergency room visits for chest pain and, in the Western hemisphere, idiopathic pericarditis (IP) is the most common form of pericarditis, where it accounts for upwards of 90% of cases [1]. Most of the evidence regarding pharmacotherapy for the treatment of pericarditis is for idiopathic (viral) pericarditis (IP) [4,5,6,7,8]. Currently, the 2015 European Society of Cardiology (ESC) Guidelines for the diagnosis and management of pericarditis, recommends combination therapy with aspirin (ASA) or a non-steroidal anti-inflammatory drug (NSAID) tapered over 3–4 weeks, plus colchicine therapy for 3–6 months, as part of first-line therapy for the treatment of IP [2]. It is important to use combination therapy, as the incidence of idiopathic recurrent pericarditis (IRP), defined as recurrence of pericarditis after a symptom free interval of 4–6 weeks, can be upwards of 50% in patients that are not appropriately treated with the combination of ASA/NSAIDs and colchicine therapy [7,8,9]. IRP results in re-admissions with associated morbidity, increased utilization of expensive healthcare resources, impaired quality of life, and can lead to complications, such as cardiac tamponade (in which pericardial fluid accumulates under pressure, impairing diastolic filling and resulting in hemodynamic collapse) [10,11]. While ASA and NSAIDs are part of first-line therapy for the treatment of IP, their benefit in the treatment is unclear, as the incidence of recurrence is higher in patients receiving ASA or NSAIDs as monotherapy, compared to those receiving ASA or NSAIDs in combination with colchicine [4,5,6,7,8]. The purpose of this review is to discuss the mechanism of ASA/NSAIDs in the treatment of IP, describe the clinical literature supporting the use of each agent, to review pertinent pharmacotherapeutic considerations including ASA/NSAID disposition, adverse effects, and monitoring and, finally, to provide outpatient and inpatient considerations for the use of ASA/NSAIDs in the treatment of idiopathic (viral) pericarditis.

## 2. Mechanism and Benefit of ASA/NSAIDs in the Treatment of Idiopathic (Viral) Pericarditis

Acute IP is thought to be potentiated by the visceral and parietal layers of the pericardium “rubbing together,” causing inflammation [1]. This inflammation can cause chest pain, which is usually worse with inspiration, and may radiate to the trapezius ridges [1]. Much less is understood regarding IRP. IRP is thought to be caused by an autoimmune phenomenon, especially in those patients who do not respond to treatment with ASA/NSAIDs and colchicine [9,12,13]. The mechanism of action of ASA/NSAIDs on IP is similarly nebulous, as unlike colchicine, ASA/NSAIDs have never been shown to ameliorate the actual disease process [2]. This may be explained by the fact that ASA/NSAIDs do not inhibit the culprits responsible for initiating inflammation—leukocytes. ASA/NSAIDs mainly inhibit the various cytokines responsible for propagating inflammation, thus improving symptoms due to the inflammatory processes [2,14,15]. In general, ASA/NSAIDs elicit their anti-inflammatory mode of pain relief by inhibiting the pro-inflammatory enzyme, cyclooxygenase (COX) [16]. COX metabolizes arachidonic acid into prostaglandins, which are important inflammatory mediators that cause hyperalgesia [16]. There are two main forms of COX, COX-1 and COX-2, both of which play an integral role in the management of pain [16]. However, both forms of COX are involved in various normal cellular processes, such as renal blood flow, gastric mucus production, platelet aggregation, inflammatory and immune processes, and vasodilation/vasoconstriction of the peripheral blood vessels [16]. Thus, the balance between agonizing and antagonizing these two enzymes is crucial in maintaining homeostasis. Large amounts of prostaglandin E_2_ (PGE_2_) are known to be released from the parietal layer of the pericardium [17]. PGE_2_ is predominant in inflammatory conditions, and potentiates nociception along with other mediators throughout the inflammatory cascade [16]. However, whether or not local COX enzymes are solely responsible for creating such prostaglandins that are present within the pericardium has yet to be elucidated.

An important concept in the analgesic and anti-inflammatory actions of ASA/NSAIDs is their ability to bind irreversibly or reversibly, as well as their specificity in inactivating COX [16]. For example, ASA irreversibly inactivates both COX-1 and COX-2, but exhibits a greater degree of COX-1 inhibition relative to COX-2 [16]. Additionally, relatively higher doses of ASA/NSAIDs are needed to elicit anti-inflammatory effects which are necessary for symptom control in IP [4,5,6,7,8,16,18]. This is most likely due to the fact that most of the inflammatory effects are mediated by COX-2, relative to COX-1 [16]. Ultimately, the role of ASA/NSAIDs in the treatment of pericarditis is to provide symptomatic relief by decreasing inflammation. The importance of mitigating inflammation is underscored by the association of elevated C-reactive protein (CRP), specifically high-sensitivity (hs)-CRP in IP [19]. CRP is considered a non-specific inflammatory marker that is elevated in many inflammatory disease states [20]. Pathologically high levels of hs-CRP may be associated with a poorer prognosis in IP [19].

Even though ASA/NSAIDs are part of the first-line therapy regimen for treatment of acute IP and IRP, only a select group of agents have been studied specifically in IP; namely, ASA, ibuprofen, indometacin, and ketorolac tromethamine. It should be noted that in many Western countries, such as in the United States, the use of ASA and NSAIDs in the treatment of IP is not approved by the Food and Drug Administration and is considered “off-label.” The following two sections will focus on the clinical literature surrounding the use of ASA and NSAIDs in the treatment of IP. It should be noted that much of the literature characterizing ASA and NSAID use in pericarditis is circumvented around the benefit of colchicine in the treatment of IP and has been previously reviewed [21]. Since there are no data directly comparing one agent to another in terms of efficacy or safety, the choice of ASA/NSAID should be dictated by the following: tolerability, cost, route of administration, pharmacokinetic/pharmacodynamic considerations, adverse effect profile, and drug-disease interactions [4,5,6,7,8]. These principles will be discussed for each agent in their respective sections throughout this review.

## 3. Aspirin Therapy for the Treatment of Idiopathic Pericarditis

ASA is rapidly absorbed orally, and analgesia increases with dose escalation [22]. During absorption, ASA is hydrolyzed to salicylic acid via esterase in the intestines and blood. Salicylic acid is the primary metabolite responsible for the anti-inflammatory properties, whereas the parent drug ASA is responsible for antiplatelet effects. The half-life of salicylic acid is dose dependent. At lower doses (up to 600 mg) the half-life is approximately 4 h, at moderate doses (1 g) it is approximately 6 h, and at higher doses (greater than 1 g), it is approximately 10 h. Therefore, when treating IP or IRP, ASA should be initiated at 600 to 800 mg every 6 to 8 h, especially for the first 7 to 10 days, and then be tapered over 3–4 weeks. Historically, the use of ASA in the treatment of pericarditis dates back to the 1980s, and it is one of the oldest documented agents used in the treatment of pericarditis [14]. At that time, ASA was used primarily in the treatment of post-myocardial infarction pericarditis (PMIP) and Dressler’s syndrome, both of which are types of pericarditis that occur after an acute myocardial infarction. To date, there is more clinical experience using ASA in the treatment of pericarditis, when compared to NSAIDs. However, it was not until 2005 that ASA was prospectively studied along with colchicine for the treatment of acute IP in the Colchicine for Acute Pericarditis (COPE) trial [4].

COPE was the first landmark study to ascertain the safety and efficacy of colchicine as an adjunct to NSAIDs or ASA for the treatment of the first episode of acute IP. Prior to COPE, limited data regarding the use of ASA or NSAIDs as first-line monotherapy for the treatment of IP existed [4]. In this single-center, open-label study, 120 patients were randomized to conventional therapy (ASA 800 mg orally every 6 to 8 h for 7–10 days then tapered over 3–4 weeks), or conventional therapy plus three months of colchicine therapy. Compared to conventional therapy, combination therapy with ASA plus colchicine resulted in a lower 18 month recurrence rate of pericarditis (32.3% *versus* 10.7%, respectively (*p* = 0.004); number needed to treat (NNT) = 5 (95% CI 3.1 to 10.0). Follow-up data also revealed that compared to ASA alone, the combination of ASA plus colchicine significantly decreased symptom persistence at 72 h and that no “severe adverse effects” were noted amongst either group. Minor adverse effects in the ASA-only group were documented in over 6% of patients and included abdominal pain and dyspepsia; these were not significantly different compared to the colchicine group. Ultimately, the COPE trial was the first study to emphasize the importance of using combination therapy with colchicine and ASA for the treatment of IP.

Published in 2013, the Investigation on Colchicine for Acute Pericarditis (ICAP) study was a multicenter, double-blind, prospective trial to assess whether or not there was a difference in recurrent or incessant pericarditis (with symptoms persisting for 4–6 weeks (that is generally the approximate length of conventional anti-inflammatory therapy and its tapering) [2], rates in 240 patients with acute IP randomized to ASA or NSAID monotherapy, or the combination of ASA or an NSAID plus colchicine therapy [7]. Similar to the COPE trial, most of the patients included in the trial were diagnosed with an idiopathic cause of acute pericarditis and in the ICAP trial, upwards of 80% of patients were randomized to ASA 800 mg orally every 8 h for 7–10 days. After 7–10 days, the doses for either ASA or ibuprofen were tapered over a period of 3–4 weeks. Results of the trial were similar to the COPE trial. However, the ICAP trial’s primary endpoint was a composite of incessant *or* recurrent pericarditis. In ICAP, the primary endpoint of incessant or recurrent pericarditis occurred in 20 patients (16.7%) in the colchicine group and in 45 patients (37.5%) in the placebo group (relative risk reduction in the colchicine group, 0.56; 95% CI, 0.30 to 0.72; *p* < 0.001); NNT= 4. Similar to the COPE trial, secondary endpoints revealed that patients randomized to ASA or NSAIDs plus colchicine therapy had significantly less symptoms at 72 h, less incessant pericarditis, fewer recurrences, and higher rates of remission compared to ASA or NSAID monotherapy. Results were similar regardless of whether the concomitant anti-inflammatory therapy was ASA or ibuprofen. No serious adverse effects were reported. GI disturbance was the main adverse effect that occurred in 2.8% of patients treated with an ASA or NSAID alone, compared to 9.2% of patients receiving colchicine.

More data are available surrounding the use of ASA in the treatment of IRP, relative to NSAIDs. Shortly after the COPE trial was published in 2005, the Colchicine for Recurrent pericarditis (CORE) trial was published the same year [5]. The CORE trial was a prospective, randomized, open-label, parallel-group study to investigate the safety and efficacy of colchicine therapy as adjunct to conventional therapy (ASA only), for treatment of the first episode of recurrent pericarditis. Upwards of approximately 85% of the 84 patients included in CORE had a previous history of acute IP. Patients were randomized to receive either ASA 800 mg orally every 6 or 8 h for 7–10 days, with gradual tapering for 3–4 weeks, or treatment with ASA at the same dose, in combination with six months of colchicine. The primary endpoint of the study was first recurrence of pericarditis and the results of the trial revealed that actuarial recurrence rates at 18 months were 50.6% in patients receiving ASA only, *versus* 24.0%, in patients receiving both ASA plus colchicine therapy (*p* = 0.02), with an absolute risk reduction of 26.6% ; NNT = 4. Similar to the COPE and ICAP trials involving patients with acute pericarditis, patients randomized to ASA plus colchicine had a significantly longer symptom-free period, compared to patients receiving only ASA. No serious adverse effects were recorded and no minor adverse effects were reported. Adverse effects were not significantly different for the ASA only group compared to the colchicine group.

Improving upon the design of the CORE trial, the Colchicine for Recurrent Pericarditis (CORP) trial was a randomized, prospective, placebo-controlled study that focused on the safety and efficacy of colchicine added to either ASA or ibuprofen for the secondary prevention of pericarditis in 120 patients [6]. Patients were randomly assigned to receive six months of placebo or colchicine in addition to ASA 800 to 1000 mg every 8 h for 7–10 days, with gradual tapering over 3–4 weeks. The results of CORP showed that over 50% of patients randomized to ASA or ibuprofen alone experienced the primary endpoint, *versus* 24% of patients who received combination therapy with ASA or ibuprofen plus colchicine (*p* < 0.001); NNT = 3. Secondary endpoints of the trial also showed a significant decrease in symptom persistence at 72 h and an increase in remission rate in those patients randomized to colchicine plus ASA when compared to ASA alone. While no severe adverse effects were noted, gastrointestinal intolerance was reported in 5% of patients randomized to ASA or NSAIDs alone, and one case of hepatotoxicity related to hepatobiliary tract disease was observed in the ASA or NSAID alone group; adverse effects for the ASA or NSAID only group were not significantly different when compared to the colchicine group.

The most recent landmark study of the efficacy and safety of colchicine for treatment of recurrent pericarditis (CORP-2), evaluated the combination of ASA/NSAIDs plus colchicine *versus* ASA/NSAIDs as monotherapy in 240 patients [8]. The CORP-2 trial was the first, and is currently the only, randomized double-blind placebo-controlled, multicenter study to assess the efficacy and safety of colchicine for the treatment of multiple recurrences (two or more) of pericarditis. Approximately 80% of patients received ASA 800 mg orally every 8 h for 7–10 days with tapering over 3–4 weeks, and 85% of patients enrolled had idiopathic pericarditis. The results of the trial showed that, among those patients who received ASA as monotherapy, 42% had continued recurrence of pericarditis, compared with 21% of patients who received combination therapy with colchicine and ASA/NSAID therapy (*p* = 0.0009); NNT = 5. Similar to previous trials, significantly more patients in the colchicine group had less symptom persistence at 72 h, a less incessant course, fewer pericarditis-related hospital admissions, and a significantly greater remission at one week, compared to ASA/NSAID therapy alone. Adverse effects that occurred in both the ASA or NSAID alone group and colchicine group were GI intolerance (diarrhea, nausea, cramping, abdominal pain, or vomiting) and hepatotoxicity. There were no significant differences between groups.

## 4. NSAIDs for the Treatment of Idiopathic Pericarditis

Those NSAIDs with the most evidence for treating IP have similar pharmacokinetic profiles [18]. NSAIDs, in general, are well absorbed, possess a relatively narrow volume of distribution, are highly protein bound, and undergo minimal first-pass hepatic metabolism [18]. NSAIDs differ in their half-life and potency. Inhibition of COX-2 expressed in endothelial cells, macrophages, and mast cells is considered the most likely mechanism of action for NSAID-mediated analgesia in pericarditis [16]. However, to date there have been no studies specifically analyzing predominant COX-2 inhibition on the inflammatory sequela of IP. As previously discussed, the majority of evidence using anti-inflammatory therapy for IP involves the use of ASA. However, the more recent landmark studies involving pharmacotherapy for IP have also included the use of agents such as ibuprofen and indometacin. The use of other NSAIDs, such as ketorolac and naproxen, are limited to case reports and cohort studies.

### 4.1. Ibuprofen

Ibuprofen is a non-selective COX inhibitor. In the United States, ibuprofen is available over-the-counter (without a prescription), and is also manufactured as an intravenous solution. Ibuprofen inhibits COX-1 approximately 2.5 times more potently than COX-2 [23]. Therefore, higher doses may be needed to produce anti-inflammatory effects. Ibuprofen is rapidly absorbed orally, with the area under the plasma concentration time curve (AUC) being dose dependent [24]. Ibuprofen’s onset of analgesia occurs within 20–60 min and its half-life is approximately 2 h [24,25]. Subsequently, frequent dosing is critical in maintaining analgesia in patients with IP. Hepatic metabolism of ibuprofen is carried out by the cytochrome P 450 (CYP450) isoenzymes 2C9 and 2C8 and yields metabolites [24]. The majority of the metabolites are then excreted primarily in the urine, as well as in feces [24]. Ultimately, the elimination of ibuprofen is dependent on urinary excretion which may be impacted by impaired renal function [24].

The use of ibuprofen for the treatment of pericarditis dates back to the 1980s [26]. It was not until 2011 that ibuprofen’s efficacy in IP was prospectively studied in patients with IRP, in the CORP trial [6]. In the CORP trial, over 90% of patients were randomized to ASA or ibuprofen. Ibuprofen was initiated at 600 mg orally every 8 h for 7–10 days, with gradual tapering over 3–4 weeks. The same ibuprofen regimen was later adopted into the ICAP trial in patients with acute IP. In ICAP approximately 24% of patients received ibuprofen 600 mg orally every 8 h for 7–10 days with gradual tapering over 3–4 weeks. Investigators of ICAP concluded that results of the trial were similar, regardless of whether ASA or ibuprofen was chosen. The CORP-2 trial also studied ibuprofen in the treatment of multiple recurrences of IRP, with approximately 15%–20% of patients receiving ibuprofen. While not the primary endpoint, an analysis of the patients receiving ASA or NSAIDs in CORP-2 revealed that there was a similar incidence of recurrent pericarditis in each group. This data supports the fact that there may be no significant clinical difference between ASA and NSAIDs in the treatment of multiple recurrences of IRP.

### 4.2. Indometacin and Ketorolac Tromethamine

Indometacin is a reversible inhibitor of both COX-1 and COX-2 that is only available with a prescription in the United States. It is available as a capsule, suppository, intravenous solution, and an oral solution. Indometacin is rapidly absorbed, with a relatively short half-life of approximately 4.5 h. It is primarily metabolized via the liver and undergoes significant enterohepatic circulation. The fact that indometacin undergoes enterohepatic circulation explains why it may only need to be dosed two to three times daily. Additionally, unlike other NSAIDs, indometacin has been found to cross the blood-brain barrier, which accounts for its significant central nervous system (CNS)-related adverse effects. Relative to the aforementioned anti-inflammatory agents, the use of indometacin for the treatment of IP has been reported sporadically in the literature [27,28,29]. In the CORP-2 trial, approximately 10% of patients were randomized to indometacin. Patients in CORP-2 were initiated on 50 mg of indometacin orally every 8 h for 7–10 days, and then tapered over 3–4 weeks. As described above, significantly less recurrence was observed in patients randomized to indomethacin plus colchicine, *versus* ASA or NSAID as monotherapy. CORP-2 was the first trial to include indometacin in the treatment of IP, specifically in patients with idiopathic recurrent pericarditis.

Ketorolac tromethamine is an NSAID that reversibly inhibits both COX-1 and COX-2. It is available in both a parenteral (IV and IM) and oral formulation. Ketorolac tromethamine is considered a potent analgesic with moderate anti-inflammatory effects. Notably, the analgesic activity of 30 mg of ketorolac tromethamine is estimated to be equivalent to approximately 12 mg of morphine sulfate, making this an attractive option for the acute management of pain [30,31]. Despite the fact that ketorolac tromethamine has been approved since the early 1990s, its use in the treatment of IP has been scarcely reported. In a pilot study by Arunasalam *et al.*, a cohort of 22 patients were given ketorolac tromethamine for pericarditis [32]. The majority of patients had post-cardiotomy pericarditis. None of the patients received combination therapy, consisting of colchicine plus ASA/NSAIDs, for treatment of pericarditis. Patients were given a single 30, 60, or 90 mg injection of ketorolac. After one hour, if patients experienced continued chest discomfort, they were supplemented with an additional 30 mg of ketorolac. Regardless of the type of pericarditis, all 22 patients responded to treatment with ketorolac and had significant reduction in chest pain with one hour. Two patients required a supplemental 30 mg dose after their initial dose. All patients were symptom free within 2 h of initiating therapy and none of the patients required additional treatment in the 36-hour follow-up period. To date, this pilot study is the only report of the use of ketorolac tromethamine in IP. Due to limited data surrounding the use of ketorolac tromethamine in the treatment of IP, clinicians must apply current recommendations when using ketorolac tromethamine for the treatment of pain associated with IP. Importantly, the total duration of ketorolac tromethamine therapy should not exceed five days due to an increased risk of serious adverse events. Therefore, clinicians must be mindful of the fact that ketorolac tromethamine is indicated only for the short-term management of moderate to severe acute pain [30]. The recommended dosing regimen for using ketorolac tromethamine for the treatment of IP is provided on Table 1. The maximum total daily dose of ketorolac tromethamine tablets is 40 mg, whereas the maximum parenteral dose is 120 mg. In patients who are ≥65 years, weigh < 50 kg, or present with moderately-elevated serum creatinine, doses of parenteral ketorolac tromethamine are not to exceed 60 mg per day.

Overall, the literature seemingly shows that colchicine is paramount in preventing recurrence of pericarditis, and that ASA/NSAIDs should be administered with colchicine to prevent recurrence of pericarditis. However, it should be noted that colchicine is not considered an analgesic [33]. ASA/NSAIDs do however provide analgesia and play a role in the management in the symptoms associated with pericarditis, such as pleuritic chest pain [2]. As previously mentioned, the symptoms of pericarditis are due to the inflammation caused by the pericardial layers “rubbing” together; this inflammation can be ameliorated by using ASA or NSAIDs. Moreover, the benefit of ASA and NSAIDs in diminishing the inflammatory sequela of IP may be quantitatively ascertained by measuring abnormal elevations in inflammatory markers, particularly with high-sensitivity C-reactive protein (hs-CRP), which can be elevated in IP [19]. The following section describes the benefits for using ASA/NSAIDs in the treatment of IP, namely their effects on decreasing hs-CRP and how this correlates with disease burden and recurrence.

## 5. Considerations When Using ASA or NSAIDs for the Treatment of Idiopathic Pericarditis

### 5.1. Tapering ASA/NSAID Therapy

In order to decrease the incidence of recurrent pericarditis, tapering of ASA or NSAID therapy has been routinely recommended. In all of the landmark trials reviewed, (COPE, CORE, CORP, ICAP, and CORP-2), the patients included in the trials were provided with an “attack dose” of ASA or NSAIDs for 7–10 days. The “attack dose” is used to decrease the acute pain associated with the profound inflammatory sequela of IP. This attack dose was then followed by a 3–4 week taper [4,5,6,7,8]. While tapering has been routinely recommended for over a decade, its importance on the prognosis and recurrence in IP was only recently elucidated with investigation into high-sensitivity CRP levels (hs-CRP) [2]. The use of CRP has been historically recommended to aid in the diagnosis and management of pericarditis. However, to date, only one prospective study has been published that ascertains patient outcomes associated with hs-CRP monitoring. Hs-CRP is more sensitive than CRP in monitoring for resolution of inflammation in IP and inexpensive commercial assays for hs-CRP are widely available in the United States [19]. In 2011, Imazio *et al.* studied the frequency of hs-CRP elevation in patients with acute pericarditis. They monitored time to normalization of hs-CRP, and its implications on diagnosis, therapy, and prognosis in IP. Hs-CRP is considered elevated at a concentration > 3.0 mg/L [19]. *De novo* hepatic synthesis exceeds 3.0 mg/dL approximately 6 h after the start of inflammation and concentrations of hs-CRP peak at 48 h [19]. From 2005–2007, 200 patients were treated with either ASA or NSAIDs for acute IP. All patients were treated with an initial seven-day attack dose of NSAIDs or ASA therapy, which was then tapered over three to four weeks. Specific attack dose regimens included: ASA 750 to 1000 mg every 8 h, ibuprofen 600 mg every 8 h, or indometacin 50 mg every 8 h. Contrary to the results of the COPE and CORE trials, colchicine was given at the discretion of the prescribing physician and not mandated. Hs-CRP was obtained at baseline and weekly until normalization was documented. The results of the study showed that 60% of patients had normalization of hs-CRP one week post-presentation, 85% of patients had normalization of hs-CRP at two weeks post-presentation, 95% had normalization of hs-CRP at three weeks, and 100% had normalization of hs-CRP at four weeks. Failure of hs-CRP to normalize at one week post presentation was found to be an independent risk factor for recurrence of pericarditis [19]. Based on the results of the study, it is recommended to initiate ASA or NSAID therapy on presentation at the attack doses listed on Table 1, with the goal of continuing attack dosing until symptoms resolve and until hs-CRP has normalized, generally within one to two weeks. While premature discontinuation of NSAID therapy during active inflammation (as evidenced by elevated hs-CRP levels) may play a role in the development of IRP, data currently remains limited. Hs-CRP monitoring may also provide optimization of symptom control and can be useful in predicting future recurrences. However, it should be noted that in approximately 25% of individuals with IP, hs-CRP is not elevated on initial presentation [19]. Therefore, a normal hs-CRP does not rule out IP as a clinical diagnosis. The 2015 ESC guidelines recommend serum CRP be obtained in order to ascertain treatment duration and response to pharmacotherapy [2]. Outpatient clinical follow-up should be arranged weekly after the initial presentation and continue until symptoms have resolved and hs-CRP levels have normalized. Once the patient is asymptomatic and hs-CRP levels are normal, then ASA or NSAIDS should be tapered every week to two weeks as long as the patient remains asymptomatic [34]. As discussed previously, currently there is no validated or accepted protocol for tapering ASA or NSAIDs in IP. However, several tapering strategies have been suggested (see Table 1) [2,35,36].

### 5.2. Safety Considerations

#### 5.2.1. Gastrointestinal

Since the COX-1 pathway produces prostaglandins that are responsible for maintaining the integrity of the gastrointestinal (GI) lining, inhibition of this process via ASA or NSAIDs can increase the risk for GI irritation and/or bleeding [37]. Since relatively high doses of the ASA/NSAIDs are needed to attenuate the inflammatory effects caused by the COX-2 pathway, GI-related adverse effects are common [38]. In order to mitigate any GI-related adverse effects associated with the use of high-dose ASA or NSAID therapy, the 2015 ESC guidelines recommend gastroprotection in all patients who receive ASA or NSAID therapy for the treatment of IP [2]. Patients who are at an especially increased risk of bleeding complications include patients on concomitant systemic anticoagulation or corticosteroid therapy, patients greater than 65 years of age, or patients with a history of peptic ulcer disease [39]. All of the landmark trials discussed above incorporated the use of a proton-pump inhibitor (PPI), (specifically omeprazole) for gastroprotection in patients randomized to either ASA/NSAIDs or ASA/NSAIDs plus colchicine [4,5,6,7,8]. Proton-pump inhibitors have a significant suppressive effect on gastric parietal cell acid production and have been found to significantly reduce the risk of NSAID/ASA-associated gastrointestinal toxicity [40]. Sucralfate has not been shown to be effective in the prevention of NSAID-induced upper GI effects. Similarly, H2-receptor antagonists are generally thought to not produce enough acid-suppression to prevent NSAID/ASA associated GI effects [41,42]. Misoprostol, a synthetic prostaglandin that helps to maintain gut integrity, is an alternate option in patients who cannot use PPI or H_2_ blockers. However, the associated increased incidence of diarrhea and abdominal pain, when misoprostol is used in combination with colchicine therapy, may limit its clinical utility for gastrointestinal prophylaxis in the setting of acute pericarditis [43].

Mild asymptomatic elevation of hepatocellular enzymes can also be seen in association with NSAID or ASA therapy, but serious hepatotoxicity is rare [44]. The rate of NSAID-induced liver injury is estimated to range from 0.29–9/100,000 patients; however, NSAIDs are thought to be responsible for 10% of total drug-induced hepatotoxicity [45]. The risk of hepatotoxicity may be increased in the setting of other hepatotoxic medications, chronic alcohol abuse, baseline liver disease, and in patients with the metabolic syndrome. The elderly may likewise be at increased risk [46]. The risk of hepatotoxicity associated with NSAIDs appears to be relatively similar across different agents, with a few exceptions. Clinicians should use ketorolac tromethamine with caution in patients with hepatic impairment or a history of liver disease. It is recommended that patients with any abnormal liver function tests be closely monitored when receiving ketorolac therapy as severe hepatic reactions, such as fulminant hepatitis, hepatic necrosis, and liver failure have occurred rarely [30]. Ibuprofen, on the other hand, has a very low incidence of liver toxicity [45]. Even so, in all patients taking chronic NSAID therapy, diligent monitoring is required, especially in patients with baseline abnormalities in liver function tests. NSAID therapy should be discontinued if signs or symptoms of liver disease manifest. ASA-induced hepatotoxicity in general is thought to be dose-dependent and may be more common in the setting of hypoalbuminemia and underlying connective tissue disease [45]. Laster *et al.* described ASA-induced hepatotoxicity in a patient treated with high-dose ASA therapy for acute pericarditis. The patient developed elevated hepatic transaminases and hepatic synthetic dysfunction within 48 h after the initiation of ASA therapy, with no other identifiable cause for hepatic dysfunction [47]. In the setting of high-dose ASA therapy for the treatment of acute pericarditis, monitoring for hepatotoxicity is, likewise, recommended.

#### 5.2.2. Cardiovascular

There are various cardiovascular safety concerns of which the clinician using ASA or NSAIDs to treat IP should be mindful. The FDA has recently issued a warning on all NSAIDs, that there is an increased risk of cardiovascular and cerebrovascular events in patients who have a history of coronary artery disease (CAD) and are simultaneously treated with NSAIDs. This risk appears to be related to the duration of therapy as well as to escalating dosage. The risk of events also appears to be increased in patients who have traditional risk factors for cardiovascular disease. In these patients, NSAIDS may contribute to adverse cardiovascular outcomes by causing unopposed propagation of COX-1 stimulation, possibly by interfering with the efficacy of ASA, by increasing the risk of GI bleeding when used in combination with ASA, by impairing scar formation post myocardial infarction, and by causing coronary vasoconstriction [48,49]. In addition, indometacin and ibuprofen have been associated with reduced coronary perfusion and increased myocardial oxygen consumption [50]. Jugdutt *et al.* found increased scar thinning after myocardial infarction in patients with PMIP treated with indometacin or ibuprofen when compared to patients who did not receive NSAID therapy [49]. Patients treated with indometacin had a greater incidence of infarction expansion syndrome compared to both those treated with ibuprofen and controls who did not receive NSAID therapy. Similar results were seen in anesthetized dogs treated with ibuprofen therapy post coronary artery occlusion [51]. With respect to ketorolac tromethamine, despite the fact that in the previously described cohort by Arunasalam *et al.*, many of the patients included in the study were treated for Dressler’s syndrome and postcardiotomy syndrome, ketorolac tromethamine is currently contraindicated for treatment of perioperative pain post coronary artery bypass graft (CABG) surgery [32]. It is thought that ketorolac tromethamine may increase the risk of myocardial infarction and stroke following CABG. Additionally, there is an increased incidence of postoperative bleeding and hematomas with ketorolac tromethamine use in the perioperative setting. ASA, on the other hand, has been historically used for the treatment of post-myocardial infarction pericarditis, post-pericardiotomy syndrome, and in patients with acute pericarditis and known vascular disease who have pre-existing indications for antiplatelet therapy. ASA therapy has been demonstrated to be both safe and efficacious in this patient population whereas NSAID therapy has been associated with increased cardiovascular risk [14]. Importantly, NSAIDs have also been shown to interfere with the antiplatelet effects of ASA. ASA exerts its antiplatelet effects by acetylating platelet COX-1 and, therefore, inhibiting platelet function. Ibuprofen has been shown to inhibit the binding of aspirin to its target site (a serine molecule on the active site of the COX enzyme), and naproxen and indomethacin have also been shown to interfere with ASA-induced platelet inhibition [52]. Therefore, in patients with a history of CAD, it is recommended to avoid the use of NSAID therapy, especially in patients with acute pericarditis in patients with a recent myocardial infarction or in those patients with a pre-existing need for anti-platelet therapy. If combined ASA and NSAID therapy cannot be avoided, the FDA recommends taking NSAIDs 30 min after taking ASA or at least 8 h prior to taking ASA to minimize drug-drug interactions [52]. The selective COX-2 inhibitors have not been studied in IP or IRP, and given the associated increased risk of cardiovascular events, including atherosclerosis, myocardial infarction, and stroke, they should be avoided.

Although variable depending on the particular NSAID, there is also a recognized association with short-term NSAID therapy and hypertension [53]. In a meta-analysis reviewing 54 studies with 123 NSAID treatment arms, there was variable effect of NSAID therapy on blood pressure. After adjustment for dietary salt intake, mean arterial pressure increased on average by 3.59 mmHg with indometacin therapy, and by 3.79 mmHg with naproxen therapy. However, ibuprofen therapy was to found to have a neutral effect on blood pressure (decrease in MAP by 0.83 mmHg), and ASA therapy was actually associated with a 1.76 mmHg reduction in mean arterial pressure [53]. The duration and time course of these blood pressure changes is unknown and variation may be due to the small sample size. Indometacin has actually been used in the treatment of orthostatic hypotension due to a hypertensive response to therapy. Kochar *et al.* treated five patients with idiopathic orthostatic hypotension with 75–150 mg of indometacin daily and found that indometacin therapy increased standing diastolic blood pressure by an average of 20–30 mmHg and resolved orthostatic symptoms in four out of the five patients. Blood pressure fell to pre-treatment levels and orthostatic symptoms resumed when indometacin therapy was discontinued [54]. Manufacturer recommendations advise that the use of ketorolac tromethamine should also be used with caution in patients with a history of hypertension, as ketorolac has been associated with new-onset hypertension or worsening of existing hypertension [30]. NSAIDs can also reduce the effects of antihypertensive therapies, especially those which are dependent on prostaglandin-mediated vasodilation [55]. While the presence of hypertension need not be an absolute contraindication to the use of NSAIDs in acute pericarditis, in individuals with hypertension, particularly those with uncontrolled hypertension on two appropriate anti-hypertensives, NSAID therapy should be minimized. If NSAIDs are chosen to treat IP, routine blood pressure monitoring is recommended. Short term courses of both low doses (100 mg) and high doses (500 mg) of ASA therapy have been associated with a neutral blood pressure effect, or a small net decrease in blood pressure. Some literature suggests that long-term use of ASA therapy is associated with increases in blood pressure. However, since ASA is generally tapered over a month, ASA-induced hypertension may be less of a concern [56]. Regardless of the anti-inflammatory treatment strategy chosen, it is prudent to also routinely monitor blood pressure in patients being treated for IP with ASA therapy.

Relative to ASA, NSAIDs have also been associated with diuretic resistance and volume retention in patients with clinical heart failure [57]. In a retrospective analysis of 10,519 patients over the age of 55, Heerdink *et al.* found a 2.2-fold increase in relative risk of heart failure hospitalization in patients on both NSAIDs and diuretics when compared to those not taking NSAID therapy (95% CI 1.7–2.9). This effect remained significant after adjustment for age, sex, and history of hospitalization (relative risk 1.8, 95% CI 1.4–2.4) [57]. Similarly, in a matched case-control study of 365 cases admitted with heart failure, compared to 658 controls, the use of NSAIDs in the previous week was associated with a doubling of the risk of heart failure hospitalization. In those patients who had preexisting cardiovascular disease, the use of NSAIDs was associated with a 10-fold increase in risk of heart failure hospitalization [58]. The effect is thought to be mediated by inhibition of prostaglandin synthesis leading to impaired peripheral and renal vasodilation, decreased circulating blood volume, as well as direct impairment of renal function. The resulting decrease in renal blood flow and glomerular filtration rate, promotes increased sodium/water retention which counteracts the effects of diuretics and can result in decompensated heart failure [59]. We would recommend using caution when administering NSAIDs in all heart failure patients. However, in those patients with escalating heart failure symptoms, poorly controlled heart failure symptoms, and in patients with intractable heart failure symptoms requiring advanced therapies (Stage D heart failure), we would recommend avoidance of NSAIDs altogether and preferentially using ASA therapy in the setting of IP.

The concern for hemopericardium is especially problematic in patients already on systemic anticoagulation in the setting of pericarditis. Presently, there is conflicting evidence as to whether or not the use of anticoagulation is a possible risk factor for the development or worsening of a hemorrhagic pericardial effusion that could potentially result in cardiac tamponade [60]. The decision of whether or not to hold anticoagulation to mitigate the risk of hemorrhagic conversion and secondary hemopericardium is controversial. Likely due to its increased time on the market relative to other anticoagulants, warfarin is the most commonly reported anticoagulant associated with the development of secondary hemopericardium in the setting of acute pericarditis. Warfarin-associated hemopericardium has been associated with serious clinical sequelae including cardiac tamponade, and even death [61,62]. Early diagnosis of warfarin-associated hemopericardium is critical, as discontinuing therapy and reversal of warfarin with fresh-frozen plasma and vitamin K, as well as pericardiocentesis can improve morbidity [62]. However, it is important to note that most of the reported cases of warfarin-associated hemopericardium were in the setting of confounders, such as trauma, cancer, drug-drug interactions, and supratherapeutic INRs [61,62,63,64]. There have also been case reports of secondary hemopericardium associated with the target-specific oral anticoagulants (TSOAs), such as dabigatran, rivaroxaban, and apixaban [65,66,67,68,69]. Both dabigatran and rivaroxaban have been reported to be associated with the development of hemopericardium in patients with concomitant renal disease as well as in the setting of possible drug-drug interactions (dronedarone and dabigatran, and saw palmetto and rivaroxaban) [66,67,68,69]. The first report of apixaban-associated hemopericardium was recently published and attributed to a possible interaction between venlafaxine and apixaban [65]. Currently, there are no published reports of edoxaban-induced hemopericardium.

Upon analysis of the available literature, it appears that the development of hemopericardium can manifest anywhere from weeks to months after the initiation of anticoagulation, and the clinician should be diligent in avoiding potentially-interacting pharmacotherapy and nutraceutical products, as well as ensuring dose adjustment in the setting of comorbid conditions, such as impaired renal and/or hepatic function. In patients that have an existing indication for chronic anticoagulation (such as atrial fibrillation/flutter and venous thromboembolism), clinicians must weigh the risk *versus* benefit of continuing anticoagulation therapy concomitantly with high-dose ASA/NSAID therapy, as anticoagulation in the setting of pericarditis is associated with a poorer prognosis and may result in an increased risk of pericardial tamponade [70]. This is especially concerning in patients receiving triple antithrombotic therapy with ASA, a P2Y_12_ receptor antagonist, and an anticoagulant, such as warfarin or the TSOAs. In these patients, clinicians should consider concomitantly administering a proton-pump inhibitor to decrease the risk of bleeding.

#### 5.2.3. Renal

Due to their inhibition of the COX-1 pathway and the subsequent impaired prostaglandin regulation of renal blood flow, there is an increased incidence of adverse renal effects in patients receiving NSAID therapy for IP. Adverse effects may include acute renal failure (albeit the absolute increased risk appears to be relatively small), acute interstitial nephritis, and rarely acute renal papillary necrosis, all of which require prompt discontinuation of NSAID therapy [71]. With the exception of ketorolac tromethamine, where administration is contraindicated in patients with advanced renal impairment and in patients at risk for renal failure due to volume depletion, in general caution is recommended with the use of NSAIDs in the setting of renal failure [30,72,73]. Although largely based on relatively small sample sizes, there are data supporting the use of indometacin in the treatment of uremic pericarditis that suggests the potential safety of indometacin in this patient population, especially in patients receiving hemodialysis. Spector *et al.*, in a prospective, double-blind trial of 24 patients with uremic pericarditis randomized to indometacin *versus* placebo in addition to conventional therapy (hemodialysis), found a significant earlier reduction in symptoms in patients taking indometacin with no noted adverse events in the indometacin group [74]. There have been several other studies, albeit also with small numbers, which likewise found that indometacin was safe and effective in the treatment of uremic pericarditis [75,76]. Given the relatively small sample sizes of present studies, definite conclusions about safety cannot be drawn, regarding the use of indometacin for treatment of IP with concomitant renal dysfunction. Clinicians should use caution if NSAIDs are chosen as first-line treatment in patients with chronic kidney disease.

Ultimately in this population, ASA is recommended as the initial treatment of choice. In a population-based cohort study by Perneger *et al.* in 1994, acetaminophen and NSAID therapy were associated with increased risk of renal disease in the general population, whereas ASA therapy did not increase the likelihood of renal disease [77]. In 2005, the UK-HARP-I Study evaluated the safety of low-dose ASA therapy (100 mg of ASA daily) *versus* placebo in patients with chronic kidney disease. There was no significant increase in major bleeding and there was no increase in progression to end-stage renal disease in the ASA group when compared to placebo [78]. As a result, ASA therapy is generally considered to be safe in patients with renal dysfunction. Overall, renal function should be assessed at baseline in patients with acute pericarditis and should be monitored during the therapeutic course. Diligent monitoring of renal function is not only important in the setting of NSAID therapy, but also because patients with IP should also be receiving concomitant therapy with colchicine, which is renally eliminated [79]. Currently, there is no official guidance on the time frame at which serum creatinine should be reassessed. Therefore, it is recommended that renal function (serum creatinine and blood urea nitrogen levels) be assessed at subsequent out-patient follow-up visits.

#### 5.2.4. Neurologic and Respiratory

NSAID therapy has been associated with a variety of adverse effects on the central nervous system including headaches, ataxia, vertigo, dizziness, nystagmus, and rarely encephalopathy, seizures, and coma [80]. The estimated risk of developing adverse neurologic effects in patients receiving NSAID therapy is approximately 5%. In general the adverse neurologic effects associated with NSAID therapy are thought to be related to inhibition of prostaglandin synthesis. Dizziness, headaches, drowsiness, and confusion are the most commonly encountered adverse neurologic effects with NSAID use [81]. Indometacin therapy in particular is associated with an increased risk of neurologic adverse effects. Indomethacin is associated with an increased risk of drowsiness, headache, and exacerbation of underlying psychiatric disturbances [72]. If patients experience neurological adverse effects while receiving a lipophilic NSAID, such as indometacin, therapy should be discontinued and either ibuprofen or ASA should be trialed.

In patients with known salicylate sensitivity or known ASA-exacerbated respiratory disease (AERD), ASA therapy is contraindicated [82]. AERD is defined as the tetrad of nasal polyps, chronic hypertrophic eosinophilic sinusitis, asthma, and sensitivity to COX-1 inhibition [82]. It is present in 0.3%–0.9% of the general population, 10%–20% of asthmatics and 30%–40% of asthmatics with nasal polyps [82]. Formal diagnosis requires an aspirin challenge. In individuals with AERD, COX-1 inhibitors induce significant upper and lower airway reactivity and can cause non-IgE mediated anaphylaxis [82]. Patients diagnosed with AERD who have not undergone ASA desensitization should avoid ASA and all NSAIDs that inhibit COX-1 [82]. Therefore, in patients with known AERD, alternative therapies for the treatment of IP (discussed below) should be recommended, as the NSAIDs used to treat IP are non-selective and generally have significant affinity for inhibiting COX-1, relative to COX-2. However, if ASA must be used, patients may undergo desensitization for ASA therapy [82]. In patients with asthma and nasal polyposis it is recommended to avoid ASA and NSAIDs until an ASA challenge has been performed

## 6. Alternative Therapies to ASA/NSAIDs for the Treatment of Idiopathic Pericarditis

While the combination of NSAIDs/ASA plus colchicine has been shown to significantly decrease the risk of recurrent pericarditis, many patients may present with contraindications to therapy due to co-morbid conditions discussed above, drug interactions, and/or intolerances. Moreover, despite adherence to combination therapy with NSAIDs/ASA plus colchicine, a subset of patients may still present with recurrent signs and symptoms of pericarditis [9]. Several strategies have been proposed to help treat patients with IP with contraindications or who are refractory to ASA/NSAID therapy [34].

Corticosteroids, namely prednisone at 0.25 to 0.5 mg/kg/day weaned over a median of five months, have been recommended for patients who present with intolerances/contraindications to ASA/NSAIDs, or who have failed combination therapy with ASA/NSAIDs plus colchicine [83]. However, corticosteroids are not routinely recommended for the treatment of idiopathic pericarditis, namely, because they have been associated with an increased risk of recurrence compared to colchicine plus ASA/NSAID therapy [4]. In the subset of patients with autoimmune-induced pericarditis that fail therapy with colchicine, ASA/NSAIDs, and/or corticosteroids, there has recently been data supporting the use of targeted immunosuppressive therapy [2,35]. Disease-modifying anti-rheumatic agents such as anakinra, azathioprine, cyclophosphamide, and cyclosporine, as well as intravenous-immunoglobulin, relatively have the most robust data regarding their safety and efficacy in the treatment of IRP that is refractory to colchicine, ASA/NSAIDS, and/or corticosteroids [35]. Nevertheless, literature surrounding the use of these agents is limited to cohorts, case reports, and meta-analysis. Before such agents can be routinely recommended for the treatment of IP, large-scale, multicenter, randomized, blinded trials need to be performed.

## 7. Summary

Despite being used as part of first-line treatment for IP and IRP, NSAIDs and ASA do not actually ameliorate the underlying inflammatory disease process that is associated with IP. Emerging research has suggested that IP may not be viral, but in fact may be due to an autoimmune processes. If this is the case, then it would explain why ASA/NSAIDs, while providing analgesia, may not actually change the underlying disease process of IP. Current guidelines recommend that, in the absence of contraindications, patients should be initiated on combination therapy with ASA or an NSAID tapered over 3–4 weeks, along with colchicine therapy for 3–6 months [2]. The efficacy of treatment should be monitored by following the patient’s symptoms, as well as objective physical findings (*i.e.*, pericardial effusion size via echocardiography, resolution of ECG manifestations, and/or resolution of pericardial friction rub). Additionally, hs-CRP should be obtained upon presentation and ideally monitored weekly for the duration of ASA/NSAID therapy. This is especially important in those patients who do not respond to an initial 7–10 days attack dose, and in patients with recurrent pericarditis. Relative to any of the NSAIDs, ASA has the most robust evidence for both the treatment of acute and recurrent IP. However, a subgroup analysis of CORP-2 revealed that there were similar proportions of recurrence of pericarditis in patients receiving ASA, ibuprofen, or indometacin therapy suggesting that there may potentially be no significant difference in effect between ASA and NSAIDs in the treatment of recurrent IP. These findings need to be further validated in a study designed to compare ASA and NSAIDs therapy in the treatment of IP from an efficacy and safety perspective [8]. For those patients who present with intolerances/contraindications to ASA or NSAIDs, corticosteroids may be utilized, and for those patients refractory to treatment with ASA/NSAIDs, colchicine, and/or corticosteroids, clinicians may consider the use of immunosuppressive therapy.

The authors report no conflicts of interest related to this manuscript. No external funding was received for the preparation of this manuscript. Dr. Schwier and Dr. Tran collectively conceived, designed, and wrote this review.

## Figures and Tables

**Table 1:** NSAIDs and ASA in the Treatment of Acute and Recurrent Idiopathic Pericarditis.

Agent	Common Attack Dose (Dose Range)	Tapering (Every 1–2 weeks)	Clinical Pearls
Acetylsalicylic acid (ASA) [2,4,5,6,7,8]	750–1000 mg PO q. 8 h (2–4 g/day)	Decrease doses by 250–500 mg every 1–2 weeks [2]	Avoid in patients with AERD Relatively safe in patients with renal or hepatic dysfunction Can be used safely in patients with HF, HTN, and/or CAD Available OTC
Ibuprofen [2,6,7,8]	600 mg PO q. 8 h (1600–3200 mg/day)	Decrease doses by 200–400 mg every 1–2 weeks [2]	Avoid in patients with AERD Available OTC Avoid in patients with HF, HTN, and/or CAD Use with caution in hepatic and/or renal dysfunction
Indometacin [2,8]	50 mg PO TID (75–150 mg/day)	Decrease doses by 25 mg every 1–2 weeks [2]	Avoid in patients with AERD Not available OTC Relatively higher prevalence of CNS-related adverse effects Avoid in patients with HF, HTN, and/or CAD Use with caution in hepatic and/or renal dysfunction
Ketorolac Tromethamine [30,32]	*No attack dose studied IM: 30–60 mg once, or 15–30 mg every 6 h IV: 15–30 mg every 6 h (max daily dose: 120 mg)	N/A	Avoid in patients with AERD Only can be used for a maximum of five days Contraindicated in patients with renal dysfunction Use with caution in patients with hepatic dysfunction Avoid in patients with HF, HTN, and/or CAD

Aspirin Exacerbated Respiratory Disease: AERD; heart failure (HF); hypertension (HTN); over-the-counter (OTC); and coronary artery disease (CAD).
